# Characteristics of Transfer RNA-Derived Fragments Expressed during Human Renal Cell Development: The Role of Dicer in tRF Biogenesis

**DOI:** 10.3390/ijms23073644

**Published:** 2022-03-26

**Authors:** Marek Kazimierczyk, Marta Wojnicka, Ewa Biała, Paulina Żydowicz-Machtel, Barbara Imiołczyk, Tomasz Ostrowski, Anna Kurzyńska-Kokorniak, Jan Wrzesinski

**Affiliations:** Institute of Bioorganic Chemistry, Polish Academy of Sciences, 61-704 Poznań, Poland; mkazimierczyk@ibch.poznan.pl (M.K.); mwojnicka@ibch.poznan.pl (M.W.); ewabiaa@gmail.com (E.B.); pzydowicz@ibch.poznan.pl (P.Ż.-M.); barim@ibch.poznan.pl (B.I.); tostr@ibch.poznan.pl (T.O.); akurzyns@ibch.poznan.pl (A.K.-K.)

**Keywords:** renal cells, tRNA-derived fragment, tRF, Dicer, Northern blot

## Abstract

tRNA-derived fragments participate in the regulation of many processes, such as gene silencing, splicing and translation in many organisms, ranging from bacteria to humans. We were interested to know how tRF abundance changes during the different stages of renal cell development. The research model used here consisted of the following human renal cells: hESCs, HEK-293T, HK-2 and A-489 kidney tumor cells, which, together, mimic the different stages of kidney development. The characteristics of the most abundant tRFs, tRFGly(CCC), tRFVal(AAC) and tRFArg(CCU), were presented. It was found that these parental tRNAs present in cells are the source of many tRFs, thus increasing the pool of potential regulatory RNAs. Indeed, a bioinformatic analysis showed the possibility that tRFGly(CCC) and tRRFVal(AAC) could regulate the activity of a range of kidney proteins. Moreover, the distribution of tRFs and the efficiency of their expression is similar in adult and embryonic stem cells. During the formation of tRFs, HK-2 cells resemble A-498 cancer cells more than other cells. Additionally, we postulate the involvement of Dicer nuclease in the formation of tRF-5b in all the analyzed tRNAs. To confirm this, 293T NoDice cells, which in the absence of Dicer activity do not generate tRF-5b, were used.

## 1. Introduction

According to current analyses, nearly 80% of the human genome has been transcribed [1]. However, only a negligible part, comprising some 20,000 genes, of which about 2% are in the form of mRNA, contain protein-coding sequences [2]. The remaining non-mRNA RNAs belong to a huge group of non-coding RNAs (ncRNAs). Due to their function, ncRNAs can be classified as housekeeping RNAs or regulatory RNAs [3,4]. Housekeeping ncRNAs, which include transfer RNAs (tRNAs), small nuclear RNAs (snRNAs), small nucleolar RNAs (snoRNAs) and ribosomal RNAs (rRNAs), are commonly expressed constitutively [5]. However, regulatory RNAs are a type of ncRNA with a strong regulatory impact on the expression of protein-coding genes. Based on their size, regulatory RNAs can be divided into two groups: small noncoding RNAs (sncRNAs), which consist of miRNAs as well as piRNAs, and long noncoding RNAs (lncRNAs) [6,7].

The main role played by tRNAs is their participation in the protein biosynthesis process. tRNAs transfer amino acids attached to their 3′ ends by a specific enzyme, aminoacyl-tRNA synthetase, to the ribosome. They then interact with the appropriate codon, embedded in mRNA, complementing it with a tRNA anticodon sequence. This guarantees that the protein has the appropriate structure and function [8]. Moreover, the translation process is dependent on regulatory RNAs, miRNAs. These are 22–23 nucleotide small RNAs obtained by cleaving longer pri-miRNAs. miRNAs bind to mRNA and influence translation mechanisms in the cell [9,10]. The role of miRNAs has been well recognized, and Fire and Mello were awarded the Nobel Prize in 2006 for their research concerning small interfering RNAs [11]. Apart from the above-mentioned roles tRNAs play in protein translation, they also have other noncanonical functions. tRNAs participate in cellular processes, such as transcription, splicing, immune responses and apoptosis, as well as acting as primers for reverse transcriptase in the retroviral genome [12,13].

In 2009, it was discovered that a second level of gene regulation involving tRNA-derived fragments (tRFs) exists [14]. The solved tRNA crystal structure showed two single-stranded regions exposed to the solution—the first, an anticodon loop, and the second, a tRNA elbow, i.e., a junction between the D and TΨC loops [15,16]. Both sites are preferred in the biogenesis of tRNA-derived fragments by specific nucleases, which generate the appropriate sets of fragments. It was suggested [17,18] that the tRFs be divided into several groups, one of which was tRF-5, which starts at the 5′ end of the mature tRNA and extends into the D loop and, at a maximum, into the anticodon stem. This variation in size allows for the further classification of tRF-5s: (i) tRF-5a extends into the D loop; (ii) tRF-5b consists of a 3′ end part of tRNA, which reaches beyond the D loop and into the D arm; (iii) tRF-5c represents the longest tRF-5 class, extending into the anticodon stem and ranging from 27 to 30 nucleotides; (iv) 5′ tRNA half; (v) 3′ tRNA half; and (vi) internal tRF. Additionally, tRFs derived from the 3′ CCA end of a mature tRNA are known as tRF-3s. These tRFs have two major size classifications: tRF-3a, which is about 18 nucleotides long, and tRF-3b, which is about 22 nucleotides long.

There are several groups of enzymes involved in tRF biogenesis. The 5′ leader sequence is a substrate for RNase P, producing the mature 5′ end of tRNA, while nuclease Z cleaves the 3′ trailer [19,20]. Moreover, it was suggested that the Dicer nuclease produces tRF-5 and tRF-3 fragments of a size similar to that of miRNA, i.e., 20–25-nucleotide long oligomers, which are cut by Dicer at the tRNA elbow end [18]. Furthermore, nuclease T2 is involved in tRF formation by cutting the D and TΨC loops in various tRNAs. [21]. Members of the RNase T2 family are found in all groups of living organisms, namely, viruses, bacteria, fungi, plants, animals, as well as humans. However, it is the tRNA anticodon loop that is the main substrate for known RNases, such as angiogenin, which is responsible for the formation of tRNA halves in mammalian cells [22].

It is interesting to note that the composition of tRFs depends on the type of organism, or even the cells present in different tissues. Moreover, the abundance of tRFs is affected by environmental factors, diet, diseases or external stimulation [23]. Therefore, a network of small non-coding RNAs—tRFs—in a cell can be an excellent regulator of cellular processes. Indeed, tRFs are known to play a significant role in various physiological and pathological processes [18,23]. Goodarzi et al. reported that in breast cancer cells, under hypoxic conditions, several tRFs were upregulated, which suppressed the stability of the oncogenic transcript by displacing its 3′ UTR from the YBX1 protein [24]. These hypoxic stress-induced tRFs can competitively bind to the YBX1 protein and block its interaction with oncogenic mRNAs. Another 3′ half tRF derived from mature tRNAGlu(UUC) has been found to be able to bind and displace the RBP nucleolin in breast cancer [25].

tRFs are involved in regulating translation during stress by competing with mRNA for ribosome binding [26]. Another way tRFs function in the cell is through their interaction with Argonaute (AGO) proteins, by forming complexes and inhibiting gene expression in the same manner as miRNAs [27]. Moreover, tRFs interact with PIWI proteins, forming stable complexes [28,29]. The immunoprecipitation of PIWIl4 (Hiwi2) from MDAMB231 cancer cells revealed the presence of the following tRFs: 5′ tRFGlu(CUC), tRFLys(TTT) and tRFVal(ACC), which are predominantly derived from processed tRNAs [28]. Similarly, the *S. scrofa* PAZ domain of the Piwil4 protein bound to the 5′ tRFVal(CAC) half [29]. Previous studies on the role of tRFs have emphasized their importance in a variety of cellular processes, including mRNA stabilization, miRNA-mediated gene silencing, as well as the regulation of cap-dependent and cap-independent translation [30]. In addition, these molecules are usually dysregulated in cancers, which is why a significant amount of research is currently focused on the role of tRFs in development and cancer [31,32,33].

## 2. Results

### 2.1. Research Model

**Figure 1:**
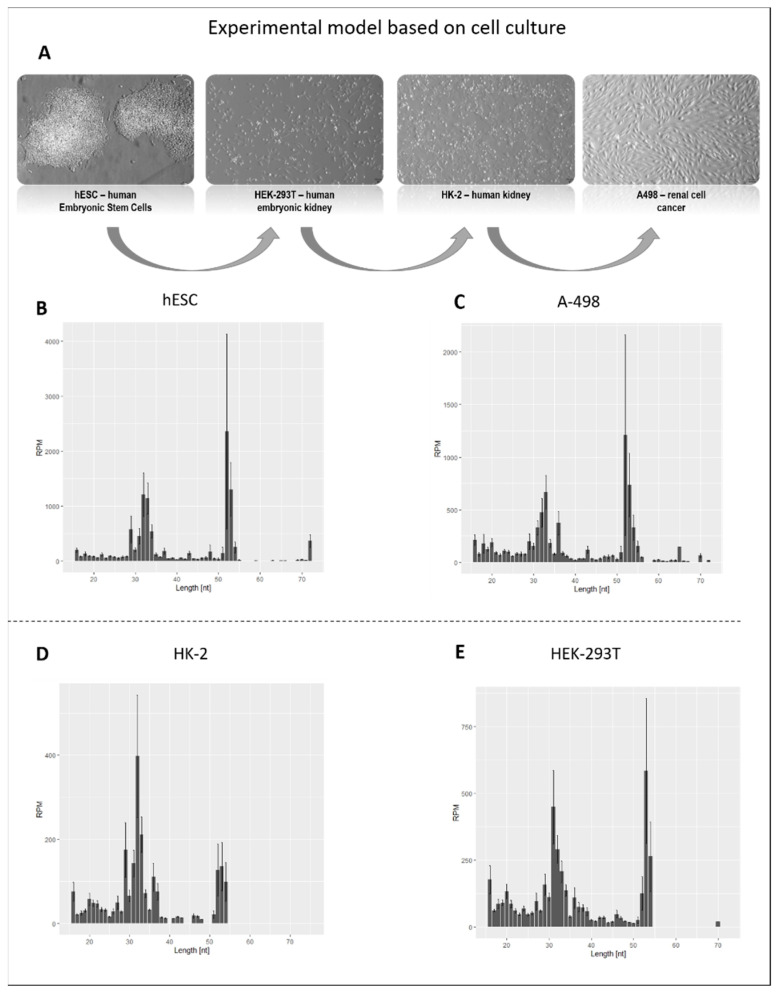
The size distribution (in nucleotide length) of tRNA-derived fragments isolated from renal cells. (**A**) The physiology of model cell cultures. (**B**–**E**) The names of the analyzed cells are shown at the top of each panel. RPM—reads per million.

Figure 1A shows the research model used to study kidney cell development. It consists of a human renal cell culture which mimics the different stages of kidney development. The first stage reflects human embryonic stem cells (hESCs), which are derived from the inner cell mass of blastocyst-stage embryos and have the ability to differentiate into various cell types. The next stage of kidney development is represented by embryonic HEK-293T cells. The original 293 cells were derived from the human embryo kidney and transformed through being exposed to fragments of sheared adenovirus 5 DNA. The HK-2 are adult cells, from an immortalized human kidney cell line. In contrast, A-498 cells, derived from a kidney carcinoma whose development was disturbed, were chosen as a model for renal cancer cells. The cells (ATCG^®^) were cultured in standard conditions, according to the manufacturer’s recommendations listed in the Material and Methods section. Figure 1, panel A presents cell culture physiology. A comparison with the manufacturer’s data shows that the cell cultures grew correctly. RNA isolated using the Trizol procedure was purified, deprived of ribosomal RNAs and fragmented. A small fraction of the obtained RNA was used for library construction. Illumina sequencing was carried out by the Macrogene company (Seoul South Korea). Bioinformatic analysis of the Illumina sequencing results revealed 30–40 million reads for each library.

### 2.2. Bioinformatic Analysis of tRNA-Derived Fragments in the Cells

Figure 1B–E display the expression of tRFs in the analyzed cells, depending on their length. It should be emphasized that there was a wide variety of tRNA fragments, with different levels of expression and length, in the model cells. tRNA oligomers, ranging from 31–35 nucleotides to 52–54 nucleotides were the most abundant. This size corresponds to cleavages in the anticodon and the TΨC loops. Moreover, there is a lower abundance of tRFs, due to the cleavage of the D loop.

It was found that the abundance of tRNA-derived fragments is dependent on the cell type. The greatest number of tRNA fragments was found in regular HK2 cells and the smallest number in hESCs (Figure 1B–E). Since multiple codons are present for many amino acids, multiple tRNAs with distinct anticodons (i.e., isoacceptors) are needed to read these codons. In humans, tRNA isoacceptors are present for 12 amino acids, ranging from two for Glu, Lys and Gln, three for Ile, Val, Thr, Ala, Gly and Pro, four for Ser and five for Leu and Arg. 

**Figure 2:**
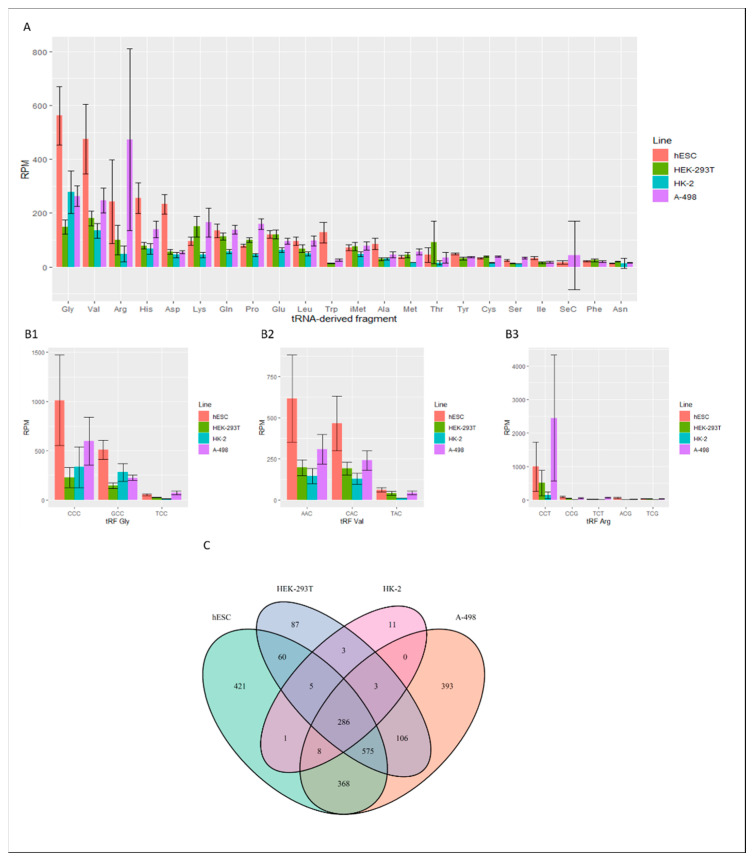
Bioinformatic characterization of tRNA-derived fragments in renal cells. (**A**) tRF occurrence in different renal cells. (**B1**–**B3**) Analysis of the most abundant tRFGly, tRFVal and tRFArg isoacceptors. (**C**) Venn diagram formed using the number of specifically expressed tRFs and their presence in different cells.

The analysis of the expression of tRFs in a selection of renal cells (stem cells, embryonic cells, adult cells and cancer cells) revealed the highest abundance of the following tRNA fragments: tRFGly, tRFVal and tRFArg (Figure 2A).

In the case of tRFHis, tRFAsp and tRFLys, their expression levels were much lower. However, in tRNAPhe and tRNAAsn-derived fragments, they were hardly expressed. Two fragments, tRFGly(CCC) and tRFGly(GCC), were the most abundant in human renal cells (Figure 2(B1)). In the bioinformatic analysis results, the highest expression of tRFGly(CCC) and tRFGly(GCC) occurred in hESC and cancer cells. However, much lower levels of expression of these tRNA fragments were found in HEK-293T and HK-2 cells during the actual experiment. The highest expression of tRFVal(AAC) and tRFVal(CAC) was noted in hESCs; by contrast, it was two times lower in cancer cells (Figure 2(B2)). The expression of these tRFs was lowest in HEK-293T and HK-2. Unlike the results obtained during the analysis, tRFArg(CCT) was the most abundant in A-498 cancer cells, while its levels were three times lower in hESCs (Figure 2(B3)). The expression of this tRF in HEK-293T and HK-2 cells was almost negligible. In Figure 2C, the Venn diagram based on the Illumina sequencing data shows the division of the identified tRF fragments into those that are specific to one type of model kidney cell and those that are found in more than one type of model kidney cell. The largest number of the identified tRNA fragments was found in hESC and A-498 cancer cells, 421 and 393, respectively. In HEK-293T and HK-2 cells, the number of tRFs present is several times lower. Interestingly, hESC, HEK-293T and cancer cells have a large number of identical tRFs—575.

### 2.3. Mapping tRFs in Renal Cells

**Figure 3:**
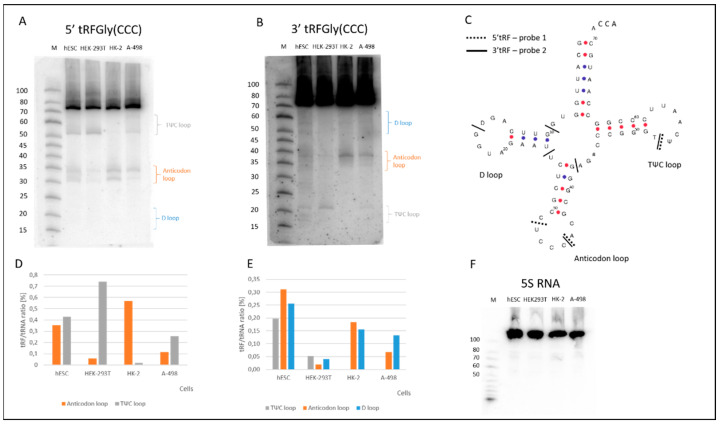
Characterization of the tRNAGly(CCC)-derived fragments. Northern blot (**A**) 5′ and (**B**) 3′ tRF detection with the use of two ^32^P labelled probes 1 and 2 respectively (Table S1). (**C**) Secondary structure of human tRNAGly(CCC), with marked 5′ (---) and 3′ (—) cleavage sites. The locations of some of the modified D (dihydrouridine), T (ribothymidine) and Ψ (pseudouridine) nucleotides are marked in the tRNA structure. The loops in the tRNA structure have been named after these modifications. (**D**,**E**) The efficiency of the tRNAGly(CCC) cleavage in loops of the analyzed renal cells. (**F**) Loading control with a hybridization probe, which matches 5S rRNA.

The results of the bioinformatic analysis were experimentally verified, using the Northern blot technique (Figure 3). Despite the fact that tRNA molecules constitute 15% of the total RNA in the cell, less than 1% of tRNA is involved in the biogenesis of tRNA fragments [34]. To detect tRFs, two 20-nucleotide long ^32^P-labeled probes were hybridized to the parental tRNA from the 5′ and 3′ ends (Table S1). For tRNAGly(CCC), three dominant cleavages in the anticodon loop, at U33, C35, generating 5′ tRNA half, and C54, generating tRF3b, were detected (Figure 3A,C). Analysis of 3′ tRF fragments detected with the use of probe 2 showed three main cleavage sites, namely, C33, C38 (3′ tRNA half) and C54 (tRF3b), with an additional one in the D loop (Figure 3B,C). Such cleavage sites indicate that all of the single-stranded regions, the D, anticodon and TΨC loops, are nuclease substrates (Figure 3A–C). When comparing the tRFs detected by both probes, both 5′ and 3′ fragments were present. There were also many fragments that were exclusively detected only by a 5′ or a 3′ probe. The first group includes tRFs, whose biogenesis takes place through the cleavage of the phosphodiester bond in the C35–C36 and T52–Ψ53 nucleotides. The second group includes 5′ tRNA halves and 3′ tRNA halves, obtained by cleavage at C31 and U34, respectively. Other tRF-5s were generated by cleavage at G10, D16 and G42. What, then, is the reason for the observed effects of the presence of one 5′ tRNA half and the absence of the other 3′ tRNA half? RNA surveillance in the cell may explain this observation, as redundant RNA is often degraded.

**Figure 4:**
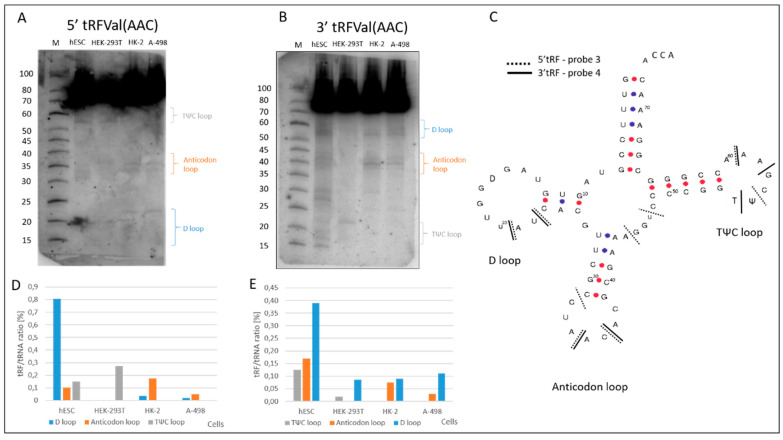
Characterization of the tRNAVal(AAC)-derived fragments. Northern blot (**A**) 5′ and (**B**) 3′ tRF detection with the application of the ^32^P labelled probes 3 and 4 respectively (Table S1). (**C**) Secondary structure of human tRNAVal(AAC) with marked 5′ (---) and 3′ (—) cleavage sites. The locations of some of the modified D (dihydrouridine), T (ribothymidine) and Ψ (pseudouridine) nucleotides are marked in the tRNA structure. The loops in the tRNA structure have been named after these modifications. (**D**,**E**) tRF occurrence and efficiency of tRNAVal(AAC) cleavage in the analyzed renal cells detected by 3 and 4 probes.

In the case of tRNAVal(AAC), the following cleavages (Figure 4A–C) were detected during tRF biogenesis. The 5′ probe 3 revealed cuts at U20 and U22 in the D loop (tRF-5b). Similar cleavages in the D loop were identified using the 3′ probe. In the anticodon loop, the most prominent cleavage occurred between A35 and A36 (5′ and 3′ tRNA halves). Additionally, cleavages at A44 and U47 (tRF-3b) were found in the variable loop (not marked), mainly in hESCs. Several cleavages were detected in the TΨC loop (Figure 4A–C). Cleavages at A58 and A60 (tRF-3b) were detected using both probes. Moreover, cleavages at T54 were most visible in HEK-293T cells.

**Figure 5:**
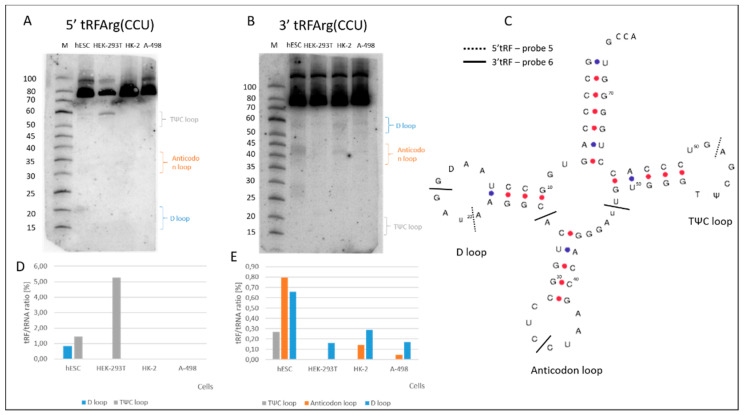
Characterization of the tRNAArg(CCU)-derived fragments. Northern blot (**A**) 5′ and (**B**) 3′ tRF detection with the application of ^32^P labelled probes 5 and 6, respectively (Table S1). (**C**) Secondary structure of human tRNAArg(CCU) with marked 5′ (---) and 3′ (—) cleavage sites. The locations of some of the modified D (dihydrouridine), T (ribothymidine) and Ψ (pseudouridine) nucleotides are marked in the tRNA structure. The loops in the tRNA structure have been named after these modifications. (**D**,**E**) tRF occurrence and efficiency of tRNAArg(CCU) cleavage in the analyzed renal cells detected by 5 and 6 probes.

tRNAArg(CCU) is the source of a limited number of fragments (Figure 4). Three different cut sites were found using probe number 5 and four sites using probe number 6 (Figure 5A–E, Table S1). It is interesting to note that there are sites that are specific to a certain cell type, such as U20 cleavage in the D loop (tRF-3b), which is specific to hESCs, just like C35 cleavage in the anticodon loop or the G51 cleavage site in the TΨC loop, which occurs only in HEK-293T cells. There are also certain kinds of cleavages which are more widespread, such as C34 cleavage (5′ tRF half), which is present in all the cells with a preference for hESCs, or G17 cleavage, which occurs in all of the model cells. 

Analysis of tRF patterns, detected by 5′ probes and 3′ probes showed that in all of the tRNAs tested here, the D, anticodon or TΨC loops were cut during tRF biogenesis, although with differing efficiency, depending on the type of cell line (Figure 3 and Figure 4). tRF-3b, formed by the cleavage of tRNAGly(CCC) in the TΨC loop at position T54, is predominantly present in hESC and HEK-293T cells (Figure 3).

Moreover, 5′ and 3′ tRNAGly(CCC) halves were almost absent in HEK-293T cells and present in other model cell cultures. In addition, a different expression of the tRF-5b fragment, obtained by cleavage of the D loop in other model cells was observed. In the case of tRNAVal(CAC), this tRF-5b occurred only in hESCs. Generally, the highest cleavage efficiency of three analyzed tRFs was detected for hESCs and the lowest for cancer A-498 cells.

The main reason why cultured A-498 cancer cells were added was to analyze the differences in the occurrence of tRFs compared to normal cells. However, the occurrence of the individual tRFs, as well as the level of their expression, was almost identical. A 50% difference was observed in the tRF/tRNA ratio for tRNAArg(CCU).

### 2.4. Is the Expression of Kidney Proteins Regulated by tRNA-Derived Fragments?

In order to answer this question, the tRFTars tool was used. [35]. In the first step, the tRF sequences detected using Northern blot analysis were entered into the tRF database and sequences with similar IDs were found. By applying the tRFTars database, potential kidney protein targets for tRF with the highest probability were found.

The presented analysis showed that the expression of different proteins can be regulated by 5′ tRFGly(CCC), 5004b (tRF-5b) and 5004c (5′ tRF halves) (Table S2A). The expression of MEIS2, FMN1 and CTDSPL2 proteins is associated with impaired renal function and can be regulated by tRF-5b, which can replace miR-458 during tumor suppression. Bioinformatic analysis of 5′ tRFGly(CCC) halves binding to protein mRNA shows their ability to regulate cellular processes, such as glucose hemostasis, the import of membrane proteins or cell energy management, while 3′ tRFGly(CCC) 3027a and 3027b can regulate the activity of a number of proteins specifically found in the kidneys (Table S2B). Seventeen-nucleotide tRF-3b can regulate Ca2+ ion transport by interacting with mRNA ORAI2 or CACNG proteins and modulating the activity of CYP20A1 and GAS7, proteins associated with kidney diseases, such as kidney nephropathy, proteinuria and chronic kidney disease.

The tRFVal(AAC)s which affect the expression of selected proteins in the kidney are limited to tRF 5017b and 3008. In silico analysis showed that tRF-5b can regulate the activity of the NFIC, GNAO1 and HIPK2 proteins, which are associated with renal tumors (Table S3A). In contrast, tRF-3b regulates the activity of the FOXD1, ARHGAP45, TOX 23 and TPCN2 proteins, which perform various functions in the development of the kidneys (Table S3B).

The role of small non-coding RNAs, mainly miRNAs, in regulating gene expression is being intensively studied. In most cases, miRNAs interact with the 3′ untranslated region (3′ UTR) of the target mRNAs to induce mRNA degradation and translational repression [8]. However, miRNAs can interact with other mRNA regions, including 5′ UTR, affecting gene expression. Does a similar mechanism of protein expression regulation exist for tRFs in the kidneys? First, a 5–7 nucleotide tRFGly(CCC) and tRFVal(ACC) seed sequence interacts with the mRNAs, presented in Tables S2 and S3, respectively. The interaction between tRF and mRNA through the seed sequence may be similar to the interaction between mRNA and miRNA. Secondly, tRFs form complexes with Ago proteins, just like miRNAs [26]. The RISC complex, consisting of miRNAs and Ago proteins, is necessary to silence protein expression [36]. However, the presented computer analysis results should be treated as preliminary, requiring experimental verification.

### 2.5. The Involvement of the Dicer Nuclease in the Generation of tRNA-Derived Fragments

**Figure 6:**
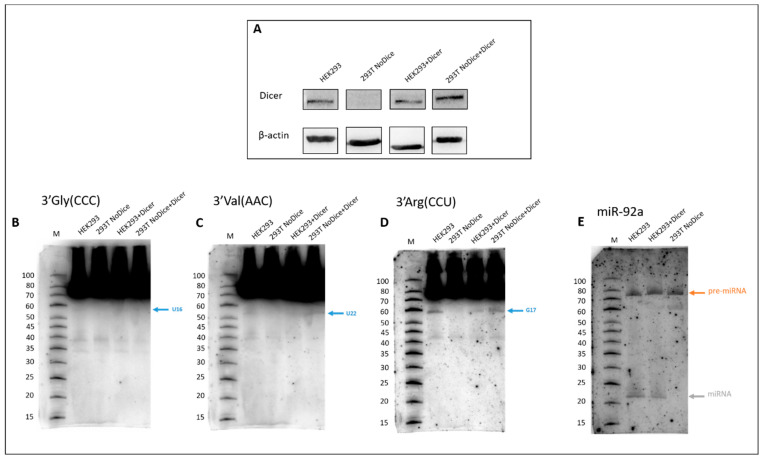
The impact of Dicer overexpression on the biogenesis of tRNA-derived fragments. (**A**) Western blot detection of hDicer nuclease expression in wild-type HEK-293T and 293T NoDice cells, before and after transfection with a plasmid producing hDicer. Cells were harvested 48 h after transfection with the plasmid producing the wild-type full-length hDicer and subsequently lysed and analyzed by Western blotting with anti-Dicer antibodies. β-actin was used as a loading control. Dicer’s influence on the cleavage of tRFGly(CCC) (**B**), tRF(AAC) (**C**) and tRFArg(CCU) (**D**). Cleavage sites in the D loop have been marked with a blue arrow. (**E**) miR-92A biogenesis in wild-type HEK-293T and 293T NoDice cells and HEK-293T cells after transfection with the plasmid producing hDicer. Pre-miRNA and mature miRNA are marked with orange and grey arrows, respectively.

Experiments demonstrating the impact of the transfection of HEK-293T and 293T (NoDice) cells with a plasmid producing full-length wild-type human nuclease Dicer (hDicer) on the efficiency of the cleavage of tRNA were carried out (Figure 6). Unlike natural HEK-293T cells, 293T NoDice cells are entirely devoid of the hDicer nuclease (the Dicer knock-out HEK-293T cells) [37]. To detect hDicer a Western blot with an anti-Dicer antibody was applied. As expected, the hDicer protein was present in the wild-type HEK-293T cells and in the cells transfected with the plasmid producing hDicer. However, the (hDicer) nuclease was not present in 293T NoDice cells. hDicer expression was only detected in these cells after they were transfected with a plasmid producing hDicer. Interestingly, the amounts of the protein produced by wild-type cells before and after they were transfected with the plasmid producing hDicer was the same (Figure 5A). This result suggests a possible mechanism that regulates the level of hDicer nuclease in the cell.

The expression of the most abundant 3′ tRFGly(CCC), tRFVal(AAC) and tRFArg(CCU) in HEK-293T, 293T NoDice and HEK-293T + hDicer, 293T NoDice + hDicer cells was analyzed, using the Northern blot approach. The efficiency of the cleavages in the anticodon loop was very similar for all of the tested tRNAs, regardless of the cell line (Figure 5B–D). However, the cleavages of these tRNAs in the D loop were strongly dependent on the cell line. For wild-type and transfected HEK-293T cells, the cleavages were almost identical, but in 293T NoDice cells, tRF biogenesis did not occur. It only occurred in 293T NoDice cells, which were transfected with a plasmid encoding the wild-type full-length hDicer. As a control, the biogenesis of miRNA 92 was used. Mature miR-92 was detected in the wild-type and transfected HEK-293Tcells but was absent in 293T NoDice cells.

## 3. Discussion

In the tested model kidney cells, many RNA oligomers are formed during the biogenesis of tRNA-derived fragments. So, then, what are the decisive factors in the biogenesis of specific tRNA fragments? One of them appears to be the expression of the parental tRNA. Indeed, it has been estimated that there is an excess of 500 tRNA genes in human cells. About half of them have been proven to be actively expressed genes [38]. Environmental conditions, such as diet and stress, influence the expression of tRFs [39,40]. Depending on the biological context, not all tRNAs are processed into tRFs, suggesting some specificity or selectivity in their biogenesis. In the human tRF database, 270 of the tRNA fragments collected had a similar number of tRF-3s and tRF-5s (tRF-3: 62, tRF-3a: 32, tRF-3b: 30, tRF-5: 63 tRF-5a: 22, tRF-5b: 23, tRF-5c: 18, 3′ and 5′ tRNA halves: 113) [41]. tRNA epigenetics is another factor, which influences tRF biogenesis. For example, the presence of 5-methyl cytidine in the tRNA chain protects it from angiogenin cleavage [42]. Recently, Guzzi et al. have observed that pseudouridine synthase 7 (PUS7)-mediated pseudouridylation is a critical regulator of the biogenesis of tRFs in hESCs [43].

The presence of the same tRNA-derived fragments in various organisms, tissues and cells has been confirmed in many reports. In epididymal spermatozoa, the expression of the three most abundant tRFs was as follows: 5-tRFGlu (34.5%), 5-tRFGly (22.0%) and 5-tRFVal (18.1%) [39]. In addition, the expression levels of 5-tRFGly, 5-tRFVal, 5-tRFMet and 5-tRFArg were significantly increased upon nutritional starvation, while 5-tRFTyr was not induced, thereby confirming the idea of specific 5-tRF biogenesis in different conditions [21]. In a recent study, tRNA halves have been identified in mouse serum using RNA-Seq [44]. The results revealed that 5-tRFGly and 5-tRFVal together account for around 90% of circulating 5-tRFs, while the majority of other 5-tRFs were below the detection limit.

The mechanism of tRF formation has not been fully elucidated. Previous studies carried out by several research groups predicted that, under stress conditions, angiogenin (ANG) cleaves tRNAs into 5′ and 3′ halves [17,45,46,47]. During normal cell homeostasis, ANG is retained in an inhibited state through its interactions with RNH1 (ribonuclease/angiogenin inhibitor 1). When the interaction is disrupted under stress conditions, it leads to ANG-mediated cleavage of the tRNAs. This observation is supported by a previous observation, that when RNH1 is knocked down, ANG-induced tRNA cleavage increases under oxidative stress conditions, indicating the importance of the ANG/RNH1 interaction in controlling the cleavage process [48]. ANG is likely not to be the only RNase to produce tRNA halves in response to oxidative stress, since they are still present in ANG knockout cells. However, additional RNase(s) await identification. ANG overexpression selectively cleaves a subset of tRNAs, tRNAGly(CCC) and tRNAVal(AAC) among them, to produce tRNA halves and tRF-5s that are 26–30 bases long. While ANG knockout has revealed that a majority of stress-induced tRNA halves, except for the 5′ half from tRNAHis(GTG) and the 3′ half from tRNAAsp(GTC), are ANG-independent, other RNases may be involved in producing tRNA halves.

The experimental results (Figure 3, Figures S1 and S2) revealed the presence of 5′ and 3′ tRNA halves in the unstressed cells. However, at the present stage of research, it is difficult to determine the role of angiogenin nuclease in cutting the anticodon loop. Angiogenin cleaves C–A and U–A sequences [49,50]. In the analyzed tRNAs, the C–A base pair occurs twice, in tRNAGly(CCC) and tRNAVal(AAC), and in both cases it is cleaved. In the remaining cases, the pyrimidine base pairs are cut. Most likely, other nucleases, such as RNase T2, are involved in their cleavage [21]. Together, the results of the present and the previous studies on human cells, yeast and plants suggest that the biogenesis of half of the tRNA requires the presence of angiogenin and/or endoribonucleases from the RNase T2 family.

Additionally, some controversy remains about the role played by Dicer proteins. Early studies of human HeLa cells have shown that the abundance of tRFGln (CUG) decreased in Dicer knockdown cells, suggesting that Dicer is necessary for tRF biogenesis [50]. However, sequencing analysis of the Dicer knockout cells in mice, *Drosophila* and yeast revealed that tRF biogenesis is independent of Dicer [46]. The results presented here show the participation of Dicer in the biogenesis of small RNAs, namely, tRFs and miRNA [51]. The abundance level of tRF-5b RNA fragments is constant in the wild-type and the transfected HEK-293T cells. Although Dicer proteins are unlikely to be major players in small RNA biogenesis, the RNase T2 family has been implicated in tRF-5 biogenesis [21]. This is suggested by the presence of cleavage sites that are not Dicer-specific. An example of this is the additional cleavage at U20 in the D loop, which was present in all of the analyzed tRNAs. 

In summary, the role of Dicer in tRF biogenesis is so far unclear (see the Introduction). Our findings showed that knockdown of the dicer gene in the case of NoDicer cells stopped the biogenesis of a 22-nucleotide long tRNA fragment of tRF-5b. This tRF appeared after the transfection of the cells with a plasmid containing the *dicer* gene. This effect is most pronounced for tRFArg(CCU) and less so for tRFGly(CCC). Perhaps Dicer is not responsible for tRF biogenesis on a global scale.

In conclusion, our research revealed that parental tRNA is a source of several tRNA-derived fragments, increasing the pool of small regulatory molecules. For example, during biogenesis, parental tRNAGly(CCC) produced 7 tRNA-derived fragments with different nucleotide lengths, tRFVal(AAC) produced as many as 11 tRFs and tRNAArg(CCU) produced 6 tRFs. The role of these tRNA fragments is unknown. Bioinformatic analysis suggests their participation in the gene expression of a number of proteins found in the kidneys by binding to mRNA in a manner similar to that of another small RNA, miRNA. However, this hypothesis requires experimental verification.

## 4. Materials and Methods

### 4.1. Cell Cultures

All the cell cultures were from ATCC (American Type Culture Collection, Manassas, VA, USA). A-498 and HEK-293T cells were cultured in Minimum Essential Medium Eagle (MEM) supplemented with 15% fetal bovine serum, non-essential amino acids (Gibco Thermo Fisher Scientific, Waltham, MA, USA), 100 U/mL of penicillin G and 0.1 mg/mL of streptomycin sulphate (Sigma-Aldrich St. Louis, Missouri, USA). The cells were cultured at 37 °C, in an atmosphere of 5% CO_2_ and 95% air, with a humidity of approximately 95%. The HK-2 cells were cultured in Keratinocyte SFM (serum-free medium) supplemented with the human recombinant epidermal growth factor (rEGF), bovine pituitary extract (BPE), non-essential amino acids (Gibco Thermo Fisher Scientific), 100 U/mL of penicillin G and 0.1 mg/mL of streptomycin sulphate (Sigma-Aldrich). The hESCs were cultured on Geltrex, in supplemented Essential 8 medium. Geltrex was mixed with cold DMEM/F12 at a 1:1 ratio, then poured onto a 60 mm plate. The prepared plate was incubated for 1 h at 37 °C. Essential 8 was supplemented with RevitaCell at a 100:1 ratio. The medium was changed every day. Passage occurred as the colonies began to grow upwards. For passage, DPBS + EDTA in a ratio of 100:1 was used instead of trypsin to detach the cells, then wash medium (DMEM/F12 and BSA in a ratio of 100:1) was used to suspend the detached cells. The cells were cultured at 37 °C, in an atmosphere of 5% CO_2_ and 95% air, with the humidity level at approximately 95%.

The 293T NoDice cells (the Dicer knock-out HEK-293T cells) [37] were cultured in DMEM (Gibco Thermo Fisher Scientific) supplemented with 10% FBS (Gibco Thermo Fisher Scientific) and penicillin–streptomycin (100 U/mL of penicillin and 100 µg/mL of streptomycin respectively, and 1 mM sodium pyruvate (Gibco Thermo Fisher Scientific), as described in Bogerd et al. [45]. For the transfection of 293T NoDice cells, the expression plasmid, including the full-length cDNA encoding human transcript variant 2 of DICER1 (NM_030621.4), was used as described previously [51]. Transfection was carried out using DharmaFECT kb DNA Transfection Reagent (Dharmacon, Lafayette, CO, USA), according to the manufacturer’s instructions. The 293T NoDice cell line was kindly provided by Prof. Bryan R. Cullen.

### 4.2. Cell Lysis and Western Blot Analysis

Cells were collected 48 h after transfection, precipitated and resuspended in lysis buffer (30 mM Hepes pH 7.4, 100 mM KCl, 5 mM MgCl_2_, 10% glicerol, 0.5 mM DTT and 0.2% Tergitol) containing 1× protease inhibitor without EDTA (Sigma-Aldrich) and broken by passing through a 0.9 × 40 mm needle. Lysates were centrifuged at 13,000 rpm for 5 min at 4 °C. Cell extracts were separated on 10% SDS-PAGE and electrotransferred onto a PVDF membrane (Gibco Thermo Fisher Scientific). For hDicer detection, the blots were probed with a mouse monoclonal primary anti-Dicer antibody mapping at the C-terminus of hDicer (1:300, Santa Cruz Biotechnology, Dallas, TX, USA); for β-Actin, the blots were probed with a rabbit monoclonal primary anti-β-actin antibody (1:1100, Cell Signaling Technology, Danvers, MS, USA) and subsequently with HRP-conjugated secondary antibody, anti-mouse or anti-rabbit (1:5000, Jackson ImmunoResearch Laboratories, Inc., Cambridgeshire, UK). The immunoreactions were detected using SuperSignal^TM^ West Pico PLUS Chemiluminescent Substrate (Thermo Fisher Scientific, Waltham, MA, USA).

### 4.3. RNA Isolation

Two different ways of isolating RNA were used. In the first, the total RNA was extracted from the culture cells using the TRIzol LS reagent (Invitrogen, Carlsbad, CA, USA), according to the manufacturer’s instructions. The concentration and the purity of the total RNA was determined using a NanoDrop™ ND-1000 spectrophotometer (NanoDrop, Thermo Fisher Scientific, Inc., Wilmington, DE, USA). To eliminate redundant modifications that could interfere with the small RNA sequencing library preparation in the subsequent steps, the total RNA was pretreated using the rtStarTM tRF and tiRNA Pretreatment Kit (cat. no. AS-FS-005, Arraystar, Rockville, MD, USA), in accordance with the manufacturer’s instructions.

The second small RNA isolation technique involved the use of the mirVana miRNA Isolation Kit, according to the manufacturer’s instructions (Invitrogen). The total RNA isolated with the Trizol method was additionally cleared using the mirVana Isolation Kit to discard long RNA and rRNA. The sRNA fraction present in the filtrate was then mixed with 100% ethanol, in the amount of 2/3 of the volume of the filtrate, and purified using a second filter cartridge. The concentration of the eluted sRNAs was measured using a NanoDrop spectrophotometer.

### 4.4. Library Construction and Illumina Sequencing

The small RNA (sRNA) fractions extracted from the cell culture were subjected to 12% (*w*/*v*) denaturing PAGE (polyacrylamide gel electrophoresis) and the sRNA fragments (18–40 nucleotides) were isolated. In the next step, the RNAs were analyzed using a Bioanalyzer 2100 with a small RNA assay (Agilent, Santa Clara CA, USA). A sequencing library was prepared with 1 mg of each sRNA sample using the TruSeq sRNA Sample Prep Kit (Illumina), according to the TruSeq Small RNA Sample Preparation Guide. The sncRNAs were subsequently ligated with RNA 39 and 59 adapters (each ligation was carried out at 28 °C for 1 h) and reverse-transcribed using SuperScript III Reverse Transcriptase (Invitrogen) at 50 °C for 1 h. The synthesized cDNA was then amplified using Phusion DNA Polymerase, indexed primers and 11 PCR cycles (98 °C 10 s, 60 °C 30 s, 72 °C 15 s) and validated with a High Sensitivity DNA Chip (Agilent). The amplified cDNA library was size-selected using electrophoresis (60 min, 145 V) in 6% Novex TBE PAGE Gel (Invitrogen). Fragments ranging in length from 140 to 190 nt, corresponding to small RNAs (15–70 nt) with both adapters, were extracted from the gel using Gel Breaker tubes (IST Engineering) and incubated in water for 3 h (RT, Rotator Mixer RM-Multi 1 (Starlab)). Then, the libraries were purified on 5 mm filter tubes (IST Engineering) and concentrated by ethanol (100%, 3.25 vol.) and sodium acetate (3 M, 1/10 vol.) precipitation. After a second validation with a High Sensitivity DNA Chip (Agilent), the libraries were quantified using a Qubit fluorometer (Invitrogen Waltham, MA, USA). All the libraries were prepared in duplicate. Sequencing was performed by Macrogen, Inc. (Seoul, Korea).

### 4.5. Bioinformatic Analysis

Quality reports were generated with FastQC v0.11.8. The reads were then subject to adapter trimming with Cutadapt. Then, they were filtered for quality using fastq_quality_filter from FASTX-Toolkit, with –q 20 –p 95 parameters, i.e., a minimum 95% of bases in a read are required to achieve a Phred quality score of 20 or higher. Individual sequence counts were determined using DESeq. Sequence mapping was performed with the bowtie tool comparing the GtRNAdb database with the RNA-Seq results. The result list was restricted to sequences of more than 10 counts using alignment2.py. Sequences were compared using the mean of the repetitions. Big data analysis was carried out using the “dplyr” library. The “VennDiagram” library was used to create the Venn diagram; the remaining graphs were made with the “ggplot” library.

### 4.6. DNA Labeling and Northern Blotting

The DNA labeling reaction mixture contained a buffer (50 mM Tris–HCl pH 7.6, 10 mM MgCl_2_, 5 mM of DTT, 0.1 mM spermidine), 10 U T4 polynucleotide kinase, 20 mCi of (γ-^32^P)ATP with an activity of 5000 Ci/mmol (Hartmann Analytic, Braunschweig, Germany) and 20 pmol of a DNA oligomer (Table S1). After an hour-long incubation at 37 °C, the ^32^P labelled products were purified using a G25 column (Sigma-Aldrich, St. Louis, MO, USA). The radioactivity level of the labeled molecules was measured using a scintillation counter. The total RNA isolated from the different cultures was separated on 12% (*w*/*v*) polyacrylamide gels, electrotransferred onto Hybond-Nylon membranes (GE Healthcare, Chicago, IL, USA), crosslinked with UV light (120 mJ/cm^2^) and prehybridized in a 2xSSC, 1X Denhard solution for 1 h at 37 °C. Hybridization of ^32^P labelled DNA probes (20 × 10^6^ cpm) was carried out at 42 °C overnight in PerfectHyb^TM^ Plus solution (Sigma). Then, the hybridization mixture was discarded, and the blot was washed in the same solution several times, until the radioactivity in the solution disappeared. Finally, the blots were analyzed using phosphor imaging screens and a FLA-5100 image analyzer with MultiGauge software (FujiFilm).

## Supplementary Materials

The following are available online at https://www.mdpi.com/article/10.3390/ijms23073644/s1.
