# Supplementary material for: Characteristics of Transfer RNA-Derived Fragments Expressed during Human Renal Cell Development: The Role of Dicer in tRF Biogenesis
Source: Int J Mol Sci. 2022 Mar 26;23(7):3644. doi: 10.3390/ijms23073644 (PMC8998818; doi:10.3390/ijms23073644)
Supplement: Supplementary file 1 [file ijms-23-03644-s001.zip › ijms-1611387-supplementary.pdf]

Supplementary material

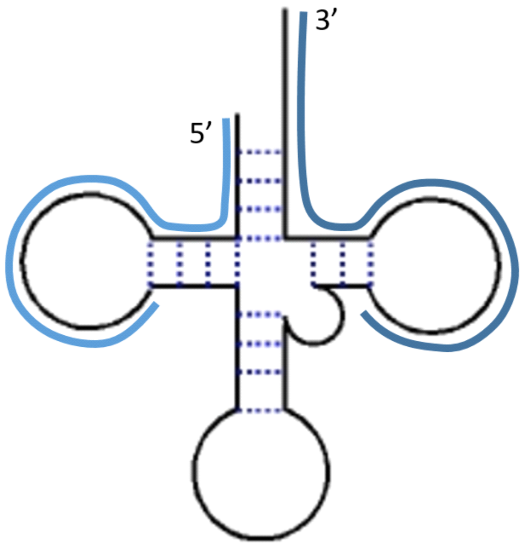

No.	Probe name	Sequence (5’ –> 3’)
1.	5'tRF Gly CCC	CGTAACCACCAAGTTACCAT
2.	3'tRF Gly CCC	AAGTTAAGGGCCGGTTACGT
3.	5'tRF Val AAC	CAAAGGCATCACATCACCAA
4.	3'tRF Val AAC	AAGCTTTGGCCCGCCTTTGT
5.	5'tRF Arg CCT	CGGGGTCACCGGATTACCTA
6.	3'tRF Arg CCT	CAAGCTCAGGGTGGACCCCAC
7.	5S RNA	AGGGTGGTATGGCCGTAGAC

Table S1 Hybridization sites of DNA probes and their sequences.

Table S2A. Computer analysis of the possibility of kidney protein regulation by 5' tRFGly(CCC)

5'tRF	tRFdb ID	Length	Positive probability	Gene	Protein	Function in the kidneys
GlyCCC	5004b	22 nt	0,8696	MEIS2	Homeobox protein Meis2	• Overexpression during aging and kidney damage [1]<br>• Participates in nephrogenesis [2]<br>• Meis1 is required for correct vascular network formation in the embryo [3]
			0,8685	SH3TC	SH3 domain and tetratricopeptide repeat-containing protein 2	• miR-584, which is located within the SH3TC intron, is a tumor suppressor, directly targeting the Rock-1 oncogene and reducing the invasion capacity in human clear cell renal cell carcinoma [4]
			0,7550	FMN1	Formin-1	• Genomic rearrangements of the FMN1 locus cause kidney defects [5]<br>• Associated with acro-renal disorders [6]
			0,7099	CTDSPL2	CTD small phosphatase-like protein 2	• Dephosphorylation of the FoxOs protein by stimulating proteolysis during nephritis [7]
	5004c	31 nt	0,8077	ZBTB20	Zinc finger and BTB domain-containing protein 20	• Glucose homeostasis [8]
			0,7730	KSR2	Kinase suppressor of Ras 2	• Cell energy homeostasis [9]
			0,7018	SEC62	Translocation protein SEC62	• Part of the ER transmembrane proteins responsible for the post-translational import

						of small presecretory proteins [10]

Table S2B. Computer analysis of the possibility of kidney protein regulation by 3' tRFGly(CCC)

3'tRF	tRFdb ID	Length	Probability	Gene	Protein	Function in the kidneys
GlyCCC	3027a	tRF3b 17nt	1,0000	ORAI2	Protein orai-2	• Transporting the Ca^{2+} ion[11]
			0,9868	CCDC9B0	Coiled-coil domain-containing protein 9B	• Cell morphology, cellular assembly and organization, cellular function and maintenance [12,13]
			0,9854	CYP20A1	Cytochrome P450 20A1	• Involved in diabetic nephropathy [14]
			0,9841	CACNG8	Voltage-dependent calcium channel gamma-8 subunit	• Involved in transporting calcium ions [15]
			0,9806	GAS7	Growth arrest-specific protein 7	• Involved in proteinuria and chronic kidney disease [16]
	3027b	22	0,9404	PLD6	Phospholipase D Family Member 6	• Member of a family of proteins correlated with Kidney Fibrosis [17]
			0,8715	LY6G6C	Lymphocyte antigen 6 complex locus protein G6c	• LY6G6C belongs to a cluster of leukocyte antigen-6 (LY6) genes located in the major histocompatibility complex (MHC) class III region [18]

1. Chang-Panesso, M; Kadyrov, F.F; Machado, F.G; Kumar, A; Humphreys, B.D. Meis1 is specifically upregulated in kidney myofibroblasts during aging and injury but is not required

for kidney homeostasis or fibrotic response. *Am J Physiol Renal Physiol*. **2018**, *315* (2), F275–F290.

2. Dekel, B; Metsuyanim, S; Schmidt-Ott, K.M; Fridman, E; Jacob-Hirsch, J; Simon, A; Pinthus, J; Mor, Y; Barasch, J; Amariglio, N; et al. Multiple imprinted and stemness genes provide a link between normal and tumor progenitor cells of the developing human kidney. *Cancer Res*. **2006**, *66* (12), 6040 – 6049.
3. Azcoitia, V; Aracil, M; Martínez-A, C; Torres, M. The homeodomain protein Meis1 is essential for definitive hematopoiesis and vascular patterning in the mouse embryo. *Dev Biol*. **2005**, *280* (2), 307-320.
4. Ueno, K; Hirata, H; Shahryari, V; Chen, Y; Zaman, M.Z; Singh, K; Tabatabai, Z.L; Hinoda, Y; Dahiya, R. Tumour suppressor microRNA-584 directly targets oncogene Rock-1 and decreases invasion ability in human clear cell renal cell carcinoma. *British Journal of Cancer*. **2011**, *104*, 308–315.
5. Dimitrov, B.I; Voet, T; De Smet, L; Vermeesch, J.R; Devriendt, K; Fryns, J.P; Debeer, P. Genomic rearrangements of the GREM1-FMN1 locus cause oligosyndactyly, radio-ulnar synostosis, hearing loss, renal defects syndrome and Cenani-Lenz-like non-syndromic oligosyndactyly. J Med Genet. **2010**, 47 (8), 569-574.
6. Natarajan, G; Jeyachandran, D; Subramaniyan, B; Thanigachalam, D; Rajagopalan, A. Congenital anomalies of kidney and hand: a review.
Clin Kidney J. **2013**, 6 (2), 144-149.
7. Liu, X; Yu,R; Sun, L; Garibotto, G; Lin, X; Wang, Y; Thomas, S.S; Li, R; Hu, Z. The nuclear phosphatase SCP4 regulates FoxO transcription factors during muscle wasting in chronic kidney disease. Kidney Int. **2017**, 92 (2), 336-348.
8. Zhang, Y; Xie, Z; Zhou, L; Li, L; Zhang, H; Zhou, G; Ma, X; Herrera, P.L; Liu, Z; Grusby, M.J; Zhang, W.J. The zinc finger protein ZBTB20 regulates transcription of fructose-1,6-bisphosphatase 1 and β cell function in mice. Gastroenterology. **2012**, 142 (7), 1571-1580.
9. Guo, L; Costanzo-Garvey, D.L; Smith, D.R; Neilsen, B.K; MacDonald, R.G; Lewis, R.E. Kinase Suppressor of Ras 2 (KSR2) expression in the brain regulates energy balance and glucose homeostasis. Mol Metab. **2016**, 6 (2), 194-205.
10. Lang, S; Benedix, J; Fedeles, S.V; Schorr, S; Schirra, C; Schäuble, N; Jalal, C; Greiner, M; Hassdenteufel, S; et al. Different effects of Sec61α, Sec62 and Sec63 depletion on transport of polypeptides into the endoplasmic reticulum of mammalian cells. J Cell Sci. **2012**, 125 (Pt 8), 1958-1969.
11. Mercer, J.C; Dehaven, W.I; Smyth, J.T; Wedel, B; Boyles, R.R; Bird, G.S; Putney Jr, J.W. Large store-operated calcium selective currents due to co-expression of Orai1 or Orai2 with the intracellular calcium sensor, Stim1. *J Biol Chem*. **2006**, *281*, 24979-24990.
12. Sassano, A; Mavrommatis, E; Arslan, A.D; Kroczynska, B; Beauchamp, E.M; Khuon, S; Chew, T.L; Green, K.J; Munshi, H.G; et al. Human Schlafen 5 (SLFN5) Is a Regulator of Motility and Invasiveness of Renal Cell Carcinoma Cells. *Molecular and Cellular Biology*. **2015**, *35*, 2684 – 2698.
13. Wang, L; Ma, S; Cui, Y; Yang, Y; Wang,D. Genes And Pathways Associated With Apparent Treatment-resistant Hypertension In Patients With Chronic Kidney Disease: An Analysis Of The Cric Study. *Hypertension*. **2020**, *76*, AP125.
14. Kiyanpour, F; Abedi, M; Gheisari, Y. A systematic integrative approach reveals novel microRNAs in diabetic nephropathy. *J Res Med Sci*. **2020**, *25*, 1.
15. Sharma, J; Deb, B; George, I.A; Kapil, S; Coral, K; Kakkar, N; Pattanaik, S; Mandal, A.K; Mavuduru, R.S; Kumar, P. Somatic Mutations Profile of a Young Patient With Metastatic

Urothelial Carcinoma Reveals Mutations in Genes Involved in Ion Channels. *Front Oncol*. **2019**, *9*, 435

16. Wuttke, M; Wong, C.S; Wühl, E; Epting, D; Luo, L; Hoppmann, A; Doyon, A; Li, Y; et al. Genetic loci associated with renal function measures and chronic kidney disease in children: the Pediatric Investigation for Genetic Factors Linked with Renal Progression Consortium. *Nephrol Dial Transplant*. **2016**, *31*, 262-269.
17. Trivedi, P; Kumar, R.K; Iyer, A; Boswell, S; Gerarduzzi, C; Dadhania, V.P; Herbert, Z; Joshi, N; Luyendyk, J.P; et al. Targeting Phospholipase D4 Attenuates Kidney Fibrosis. *J Am Soc Nephrol*. **2017**. *28*, 3579-3589.
18. Mallya, M; Campbell, R.D; Aguado, B. Transcriptional analysis of a novel cluster of LY-6 family members in the human and mouse major histocompatibility complex: five genes with many splice forms. *Genomics*. **2002**, *80*, 113-123.

Table S3A. Kidney protein regulation by 5'tRFVal(AAC)

5'tRF	tRFdb ID	Length	Probability	Gene	Protein	Function in the kidneys
Val(AAC)	5017b	tRF-5b<br>20nt	1,0000	NFIC[1]	Nuclear transcription factor	• NFIC may regulate FABP7 expression in RCC [1]
			0,9985	GNAO1	Guanine nucleotide-binding protein G(o) subunit alpha	• GNAO1 are likely to be involved in the regulation of RCC through G protein signal transduction [2]
			0,9984	HIPK2	Homeodomain interacting protein kinase 2	• Regulator of transcription factors, modulates growth, development, morphogenesis, and cell death in renal cells [3]

Table S3B. Kidney protein regulation by 3'tRFVal(AAC)

3'tRF	tRFdb ID	Length	Probability	Gene	Protein	Function in the kidneys
Val(AAC)	3008	tRF-3b<br>17nt	0,9946	FOXD1	Forkhead box protein K1	• Foxd1 is required during kidney development [4]<br>• Inactivation results in the failure of nephron progenitor cell differentiation
			0,9821	ARHGAP45	Rho GTPase-activating protein 45	• Expression of ARHGAP24 results in inhibited cell proliferation [5]<br>• Reduces cell invasion ability<br>• Induces apoptosis in renal cancer cells.

			0,9394	TOX23	TOX high mobility group box family member 2	• The genes are linked to biological pathways involving solute transport and renal physiology [6]
			0,9393	TPCN2	Two pore calcium channel protein 2	• Tpcn2 mRNA expression was the highest in kidneys, liver and adipose tissue [7]

1. Takaoka, N. Analysis of the regulation of fatty acid binding protein 7 expression in human renal carcinoma cell lines. *BMC Mol. Biol*. **2021**, 12, 31.
2. Liu X, Wang J, Sun G. Identification of key genes and pathways in renal cell carcinoma through expression profiling data. Kidney Blood Press Res. 2015;40(3):288-97.
3. Fan Y, Wang N, Chuang P, He JC. Role of HIPK2 in kidney fibrosis. Kidney Int Suppl. 2014, 4, 97-101.
4. Fetting JL, Guay JA, Karolak MJ, Iozzo RV, Adams DC, Maridas DE, Brown AC, Oxburgh L. FOXD1 promotes nephron progenitor differentiation by repressing decorin in the embryonic kidney. Development. 2014 Jan;141(1):17-27.
5. Xu G, Lu X, Huang T, Fan J. ARHGAP24 inhibits cell cycle progression, induces apoptosis and suppresses invasion in renal cell carcinoma. *Oncotarget*. 2016;7(32):51829-51839.
6. Sima, C., Iordache, P., Poenaru, E., Manolescu, A., Poenaru, C., Jinga, V. Genome-wide association study of nephrolithiasis in an Eastern European population. *Int Urol Nephrol.* 53, 309–313 (2021).
7. Tunn RE Expression of two-pore channels in mammalian primary cells and tissues, and their role in adipose tissue formation and function. eTHOS 2012
